# The DAWN antivirals trial: process evaluation of a COVID-19 trial in general practice

**DOI:** 10.3399/BJGPO.2023.0109

**Published:** 2024-04-17

**Authors:** Dajana Tare, Samuel Coenen, An De Sutter, Stefan Heytens, Dirk Devroey, Laetitia Buret, Birgitte Schoenmakers, Nicolas Delvaux, Jan Y Verbakel, Kris Bogaerts, Ann van den Bruel

**Affiliations:** 1 Academic Centre for General Practice, KU Leuven, Belgium; 2 Centre for General Practice, Department of Family Medicine and Population Health (FAMPOP), University of Antwerp, Antwerpen, Belgium; 3 Department of Public Health and Primary Care, Ghent University, KU Leuven, Belgium; 4 Primary Care, Vrije Universiteit Brussel, Ixelles, Belgium; 5 Department of General Medicine, University of Liège, Liège, Belgium; 6 Department of Public Health and Primary Care, I-BioStat, KU Leuven, Leuven, Belgium

**Keywords:** COVID-19, antiviral, randomized controlled trial, primary healthcare

## Abstract

**Background:**

The DAWN antivirals trial was a multicentric, randomised placebo-controlled trial evaluating antiviral medication for COVID-19 in general practice. The trial was prematurely terminated because of insufficient recruitment.

**Aim:**

To explore which factors contributed to the premature termination.

**Design & setting:**

General practice in Belgium.

**Method:**

Patients were randomised to camostat or placebo (patients and physicians blinded) between June 2021 and July 2022; a third arm evaluating molnupiravir (open label) was opened in March 2022. The outcome assessor was blinded for all comparisons except for the patient reported outcomes in case of molnupiravir. The authors analysed available trial data and evaluated trial context, implementation, and mechanisms of impact based on semi-structured interviews with trial stakeholders.

**Results:**

The trial recruited 44 participants; 19 were allocated to camostat (median age 55 years), 8 to molnupiravir (median age 60 years), and 17 to placebo (median age 56 years). There were no serious adverse events in either group. Most difficulties were related to the pandemic context: disruption to routine clinical services; multiple changes to the service model for COVID-19 patients; overwhelmed clinical staff; delays of trial medication; and staff shortages in the sponsor and clinical team. In addition, regulatory approval processes were lengthy and led to additional study procedures. It was felt that the trial started too late, when vaccinations had already begun.

**Conclusion:**

The DAWN antivirals trial was stopped prematurely. Although many barriers were related to the pandemic itself, hurdles such as a small and inexperienced sponsor and clinical teams, delays in regulatory processes, and research capacity in routine settings could be overcome by established research infrastructure and standardisation of processes.

## How this fits in

Treating COVID-19 patients in the community may avert hospital admissions and death, yet most therapeutic trials were conducted in hospital settings. The DAWN antivirals trial aimed to evaluate two types of antiviral medication but failed to recruit the target sample size and was therefore stopped prematurely.

The process evaluation of the trial identified several challenges and barriers, including the pandemic itself, staff shortages, difficulties in obtaining the investigational medicinal product (IMP), changing disease severity after successful vaccination campaigns, limitations of the traditional recruitment model in general practice, and the research ecosystem’s lack of expertise of running high-risk trials in general practice. Improved research infrastructure in general practice, as well as better preparedness at the political, ethical, administrative, regulatory, and legal level, may overcome some of these barriers.

## Introduction

The arrival of the SARS-CoV-2 virus in Belgium in March 2020 marked the start of a nationwide epidemic that, by 1st June 2021, would account for 1 062 001 confirmed cases, 74 149 hospital admissions, and 24 955 fatalities.^1^ Compared to other European countries, Belgium was hit particularly hard by the first and second waves.^2^ Similarly to other countries, mortality and morbidity were especially high in older age groups (65 years and older), accounting for over 90% of all COVID-19-related deaths from March 2020 to February 2021.^3^

Earlier in the pandemic, COVID treatments mostly focused on patients admitted to hospital, suffering from serious COVID-19 infections. As a result, the COVID-19 research landscape consisted of a large number of hospital-based clinical trials with only a few trials focusing on outpatients. Nonetheless, important gains could be made from treatment in the community, to prevent patients from progressing to serious illness.

In this article, the authors reported a process evaluation of the DAWN antivirals trial, which tested two antiviral drugs but was terminated prematurely because of insufficient recruitment. With this process evaluation, this study aimed to capture lessons learnt and improve future national trial set-ups in general practice.

## Method

The DAWN antivirals trial was a phase III, randomised placebo-controlled trial of antivirals in moderately ill COVID-19 patients presenting to general practice (clinical trials ID NCT04730206). The aim was to find a repurposing drug that would be suitable for widespread use in COVID-19 patients treated in general practice. The trial was funded by KCE Trials, a national funding body, and sponsored by the KU Leuven in collaboration with four other university groups across Belgium. Preparations for the study started in the third quarter of 2020, and the study opened for recruitment in the second quarter of 2021.

Drugs under investigation included camostat (June 2021–July 2022) and molnupiravir (March 2022–July 2022), both identified as drugs of interest by the national COVID-19 therapeutic task force. Camostat is a synthetic trypsin-like serine protease inhibitor that blocks the TMPRSS receptor used by the coronavirus to enter the host cells.^4^ Camostat was selected for this trial based on this in vitro effect and its excellent safety profile^5^ at a daily dose of 800mg (200mg four times a day), for 7 days. Camostat was approved in Japan in 1985 for the treatment of chronic pancreatitis and oesophagitis, but does not have market authorisation in Europe. Purchasing camostat from the Japanese manufacturer was not possible, and camostat and matching placebo tablets were therefore manufactured in India.

Molnupiravir is an oral prodrug that has shown antiviral activity against SARS-COV-2.^6^ It received a positive advice from the European Medicines Agency in November 2021.^7^ The Belgian government purchased an amount of molnupiravir, but the National Task Force for COVID-19 Therapeutics advised it should be evaluated in the DAWN antivirals trial first before allowing more routine use. The molnupiravir tablets were commercially manufactured and therefore identifiable by the Merck Sharp & Dohme logo. The dosage as accepted by the European Medicines Agency is 4 capsules of 200mg, taken twice daily for 5 days (total daily dose of 1600mg).

The control arm received a placebo. The placebo tablets were identical in size and appearance to the camostat tablets, thus blinding patients and physicians for the camostat versus placebo comparison; patients and physicians were unblinded for the molnupiravir versus placebo comparison as the molnupiravir tablets did not match the placebo tablets. The outcome assessor was blinded for all comparisons (except for the patient reported outcomes in case of molnupiravir).

Community-dwelling adults aged at least 50 years old, presenting to general practice with COVID-19 suggestive symptoms for a maximum of 5 days and a positive test for COVID-19 were eligible for the trial. The age cut-off was lowered to 40 years of age to increase the number of eligible patients. Only tests conducted by a healthcare professional were allowed.

The primary outcome was initially hospitalisation or death within 30 days after randomisation. Because of declining hospitalisation and mortality rates as a result of vaccination, a second primary endpoint was added in April 2021, namely time to first self-reported recovery within 30 days after randomisation. In March 2022, the composite endpoint hospitalisation or death was downgraded to a secondary endpoint and time to first self-reported recovery was the only primary endpoint from then onwards.

At the start, the required sample size was 1316 patients (653 in each treatment arm), to be accrued over 15 months to have 90% power on hospitalisation and/or mortality rate (odds ratio of 0.6). Randomisation was stratified by age (at least 65 years old versus younger) and comorbidities (at least three comorbidities or more versus less than three). In March 2022, due to the change in endpoint and the addition of a third arm evaluating a second antiviral drug, the required sample size was reduced to 463 patients (150 per arm, plus 13 patients already recruited) to have 90% power on time to recovery (hazard ratio of 1.5).

**Figure 1.**
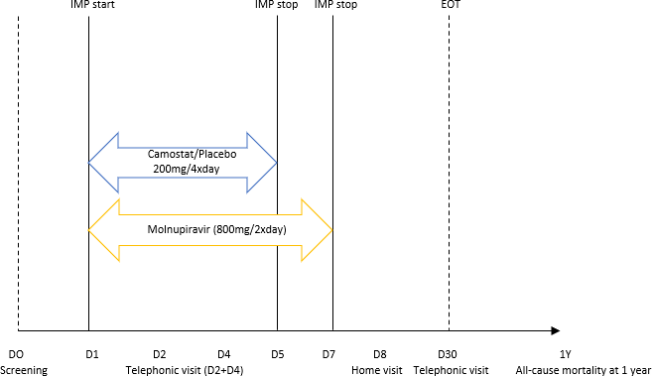
Trial related procedures.

There were five study visits: a baseline visit, a telephone visit on day 2 and day 4, a home visit on day 8, and a final home visit on day 30. Follow-up information (up until 30 days after randomisation) was requested from the participants’ regular GP. Participants were requested to keep a diary for 30 days to monitor symptoms, side effects, and compliance. Collection of mortality data at 1 year after randomisation was planned (Figure 1).

### Data collection and analysis

For this process evaluation, the authors analysed available trial data, including baseline characteristics of the included trial population, safety data, and a descriptive presentation of the primary and secondary outcomes.

Given the premature trial termination, trial data were reported descriptively without any testing. In addition, no subgroup analyses were performed and no correction for the stratification factors was applied. Continuous variables were summarised by treatment group by the number of non-missing data points, mean, standard deviation, median and interquartile range. Categorical and ordinal variables were summarised by treatment group by observed frequencies and percentages relative to the total number of non-missing items.

Secondly, the authors conducted an interview study to explore the perspectives of all parties involved in the trial, including participants of the DAWN antivirals trial, members of the trial steering group, the funding body, the study monitors, pharmacovigilance, principal investigators (PIs), as well as study nurses. The trial participants were purposively selected based on age (≥65 years, or younger), sex, their PI, and study nurse.

Semi-structured interviews were conducted by one researcher (DT) between November 2022 and January 2023. DT is a researcher with a background in physiotherapy and was involved in the trial as a trial coordinator. The goal of the interview was explained at invitation and repeated at the start of each interview. Trial participants were invited for participation by their study nurse, under the supervision of the trial PI; the other stakeholders were invited by DT. All potential participants were invited by email or telephone. The interview guide (see Appendix 1) was designed by consensus discussion between DT and AVDB and revised based on new insights from previous interviews. Interviews were conducted either online or in person. All interviews were audio-recorded, transcribed verbatim, and analysed independently. Notes were taken during the interviews to facilitate data analysis. Transcripts were not sent to participants for review.

An inductive framework analysis approach was used to analyse data. One researcher (DT) independently developed an initial coding framework used for analysis that was revised after eight interviews. The remaining interviews were then analysed using this framework by DT, while changes and additions were made when other themes emerged. Data collection was stopped when saturation was achieved (no new themes emerged in the last two interviews).

## Results

### Results of the DAWN antivirals trial

In total, 44 participants were recruited in the DAWN antivirals trial between June 2021 and July 2022; 19 were randomised to camostat (median age 55 years), 8 to molnupiravir (median age 60 years), and 17 to placebo (median age 56 years). Obesity and hypertension were the most prevalent comorbidities (Table 1). All participants were vaccinated. During the course of the trial, circulating variants were Delta (from May 2021) and Omicron (from November 2021). The mean oxygen saturation level was >97% and mean temperature was <37°C in all groups.

**Table 1.** Co-morbidities of the trial participants

	Camostat (*n* = 19)	Molnupiravir (*n* = 8)	Placebo (*n* = 17)
Diabetes type I	1	1	1
Diabetes type II	2	2	1
Hypertension	8	3	6
Heart conditions	4	1	1
Asthma	2	2	1
COPD	0	0	0
Cystic fibrosis	0	0	0
Pulmonary fibrosis	0	0	0
Obesity	5	3	4
Cancer	0	0	1
Solid organ transplant	0	0	0
Neurological	0	0	0
Thalassaemia	0	0	0
Sickle cell	0	0	0
Renal function impairment	0	0	1
Liver function impairment	1	0	0

COPD = chronic obstructive pulmonary disease.

The median number of days until self-reported recovery within 30 days after randomisation was 7.5 (95% confidence interval [CI] = 5 to 11) in the camostat group, 23.5 (95% CI = 2 to undefined) in the molnupiravir group, and 10 (95% CI = 5 to 19) in the placebo group. On day 8, *n* = 11/19 of participants in the camostat group, *n* = 6/8 of participants in the molnupiravir group, and *n* = 12/16 participants in the placebo group were still symptomatic according to the World Health Organization clinical progression scale. On day 30, this had decreased to *n* = 4/19 of participants in the camostat group, *n* = 4/7 of the molnupiravir group, and *n* = 5/16 of the placebo group.

During follow-up until 30 days after randomisation, there were no cardiovascular or thromboembolic complications, no hospital admissions and no deaths in either group. Two participants in the camostat group and one participant in the placebo group reported a hospital visit without admission. Two participants (both in the camostat group) used antibiotics.

There were no serious adverse events in either group. There were 51 non-serious adverse events in 13 participants in the camostat group, 84 adverse events in eight participants in the molnupiravir group, and 98 adverse events in 14 participants in the placebo group. The five most common adverse events were fatigue (*n* = 20), cough (*n* = 12), constipation (*n* = 10), nasopharyngitis (*n* = 10), and diarrhoea (*n* = 9).

### Process of the DAWN antivirals trial

#### Regulatory authorities

The DAWN antivirals trial was the first trial in general practice in Belgium using an unregistered product. For this reason, regulatory authorities were reluctant to grant approval and a pre-submission advice process was deemed necessary, which took more than 4 months. At the regulatory authorities’ request, telephone visits were added on day 2 and day 4 after randomisation. Regional PIs were also installed because assessing adverse events by individual GPs was deemed insufficiently reliable.

#### Trial trajectory

The study gained regulatory approval from the Ethical Committee and Competent Authority on 17 March 2021 and the first participant was recruited on 21 June 2021. Overall, five major protocol amendments were submitted: 1) lowering participant age to 40 years old and adding self-reported recovery as a co-primary endpoint 2) adapting the exclusion criterion of an earlier positive PCR or rapid test 3) adding the requirement of at least two symptoms and allowing remote informed consent 4) adding molnupiravir as a third arm; and changing the primary endpoint to self-reported recovery only with lower sample size and 5) website adaptation to facilitate self-expression of interest by potential participants.

**Figure 2.**
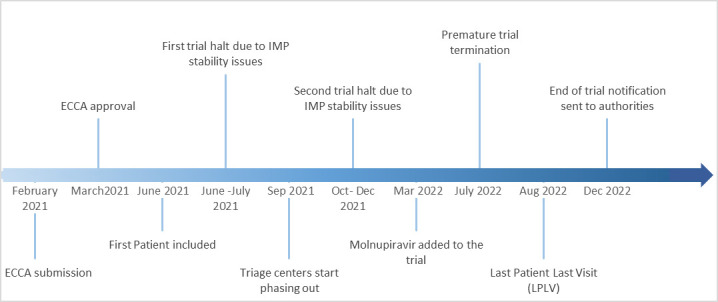
Trial trajectory. ECCA = Ethics Committee Competent Authority. IMP = investigational medicinal product.

Trial medication was delayed by 3 months because of the pandemic situation in India, labelling was not compliant, necessitating relabelling on arrival in Belgium, and documentation on 3-month and 6-month stability was unclear, resulting in temporary halts of the trial (July 2021 and September–November 2021) for independent stability testing. The trial history is displayed graphically in Figure 2.

#### Recruitment

##### Sites

At the start, testing of potential COVID-19 patients was centralised in testing centres rather than in general practices. For this reason, the authors opened the trial in four testing centres across the country and PIs were GPs working at these testing centres supported by study nurses. Over the summer of 2021, testing centres were gradually phased out and general practices became the primary recruitment setting. Eight PIs stopped participation and 10 new PIs were added to the trial in December 2021, bringing the total to 10 PIs in Flanders, three in the Walloon region, and one in Brussels. PIs were paid 50€ per recruited participant. Generally, no ancillary staff is present in general practices to support PIs with recruitment in Belgium.

##### Decentralised model of recruitment

By September 2021, remote informed consent was allowed in Belgium and the trial subsequently adopted a remote recruitment model. Study nurses visited potential participants at home while the PI conducted the informed consent procedure via video call, as requested by the regulatory body for safety reasons. A dedicated website was created as a means for potential participants to express interest in the trial. A targeted social media campaign was set up to create awareness among the public; emails, and printouts of flyers and posters were sent to more than 3000 GPs in Flanders; and pharmacies were asked to refer COVID-19 patients to the trial. Finally, news articles in the lay press and professional press aimed at increasing awareness of the trial among the public and GPs. A request to the governmental contact tracing centres to send an automatic text message with trial-related information to all confirmed COVID-19 patients was declined.

##### Recruitment rates

Over the course of the DAWN antivirals trial, 129 patients expressed potential interest in participating in the trial. Of those, 65 were screened for participation and 44 were enrolled. Reasons for not proceeding to the screening stage included loss of interest or ineligibility based on the provided information. In screened potential participants, the two main reasons for non-enrolment were: a) symptoms for longer than 5 days and b) not willing to take medication. During recruitment at the triage centres, only seven participants were included, which equals approximately one per month. The remote recruitment model increased recruitment to approximately seven per month.

Belgium has two main regions: Flanders (Dutch speaking) and the Walloon region (French speaking). Since the study was funded by a national funding body, it was mandatory that the trial ran in both regions. For this reason, the authors recruited PIs in both regions and translated all patient-facing materials in both languages. Recruitment was challenging in both regions, but more so in the Walloon region. In fact, only one participant was recruited in the Walloon region compared to 43 in Flanders.

### Study team

The sponsor team consisted of a coordinating investigator, a part-time project manager and a trial coordinator; the other two did not have experience with general practice trials. External collaborators were sought for data management and safety management. In addition, the authors contracted an external party for the IMP production, a qualified person for IMP release and IMP storage, and an external company for IMP stability testing.

The clinical team consisted of the PIs, the regional PIs, and the study nurses. Due to the very high demand for nurses during the pandemic, study nurses had to be outsourced from temporary staffing agencies at very high hourly fees. More than half of the PIs (*n* = 16) and study nurses (*n* = 4) did not have prior trial experience.

### Results of the qualitative interview study

From 25 November 2021 until 19 January 2022, 51 potential participants were invited, of whom 21 accepted and were interviewed: 4 PIs, 5 trial steering group members, 5 trial participants, 3 external trial collaborators, 2 study nurses, and 2 staff from the funding body. No pharmacists or GPs from the Walloon region accepted the invitation for an interview.

On average, the interviews lasted 35 minutes. Based on the analysis of the interview transcripts, different themes and subthemes emerged, which were then classified into three main domains: 'pandemic context', organised in four themes (Table 2); implementation, organised in five themes and nine subthemes (Table 3); and mechanisms of impact, organised in two themes and five subthemes (Table 4).

**Table 2.** Themes for domain 'pandemic context'

Themes	Illustrative quotes
Rapidly changing context	'*Right from the beginning it was clear that it was a very, very challenging project. A study with initially one drug that was not registered in Europe, implying higher risk, higher complexity — a pandemic study with inherent risk and complexity.*' [P18] '*I think that the biggest stumbling block for the DAWN trial was the constantly changing context of the infection rates, which were very unpredictable and caused that we never reached our target sample.*' [P4]
Timing	'*Even from quite early on, you know, it seemed to be a little bit late or a little bit behind the curve, so missing things, and I think it was also difficult because quite a few things changed. So, each time you make a change, a lot of effort is going into amendments to changing things. And therefore this is taking resources and your attention off getting patients in.*' [P18] '*It was bad timing for the study to start when it did. I think that if it would have started a year or six months earlier, it would have made a big difference.*' [P11] '*It’s difficult when you're up against time pressures when everything’s spread a little bit thin because of the resourcing, it’s very difficult to advance everything all at once. So, with hindsight I think piloting could have been, you know, the best way to go. I think piloting the different aspects, the different logistical aspects in a smaller region would have been a very good idea.*' [P18] '*I think having to have the drug made in India, and the issues related to that and then you know you have a delay and therefore you're missing a certain wave.*' [P13]
Care delivery during the pandemic	'*It was obvious that was really going to be a huge operational challenge to get those test centres on board and up and running to recruit*.' [P3] '*The pandemic disrupted the normal way of working. With the introduction of teleconsultations, I found it difficult to pitch the study to a patient.*' [P12] '*Recruitment via the GPs is not a bad channel because then you have some confidence about the study. I think it is a good channel, but the channel was overloaded during COVID-19.'* [P15] '*Personally I do find it difficult to approach the patients about that. I don't want to make them feel obligated to participate or anything like that to do that. I guess this is something I should work on for future studies…*' [P20]
Public opinion	'*People were fed up with COVID. Also, by that time they were not getting so sick, so they were not willing to take new medication.*' [P7]

**Table 3.** Themes and subthemes for domain 'implementation'

Themes and subthemes	Illustrative quotations
**Trial design**	
Strengths and limitations	'*The fact that it was a national study, spread across Belgium, giving the opportunity to many patients to participate, despite the COVID-19 situation back then.*' [P4] '*A broad network of healthcare professionals of general practice involved, as well as the involvement of academic centres was a very strong point of the protocol set up.'* [P5] '...*looking back on it, there were a lot of really innovative aspects, the more decentralised components, the support from the study nurses, the fact that the patients were not recruited by their own GP...'* [P18]
	'*I do not think that the limitations are linked to the protocol. I think the limitations were inherent to the overall situation…*' [P8] '*A protocol-related limitation is the fact that the whole protocol is written having the site as a central component of recruitment; a site which takes over coordination, where a number of study staff is linked to this site. This idea is built based on the "hospital principle", but this is not how it works in general practice…*' [P5]
**Trial-related procedures**	
Trial initiation	'*COVID created an extra burden for us, especially for older GPs who were against the digitalisation, having the training online did not facilitate me as much as a face-to-face training would have.*' [P9] '*There was too much time between finding and preparing a test centre/GP practice to participate in the study, getting the agreement to cooperate and then actually being able to start. There have been a lot of "false" starts, so that many test centres and doctors were not so motivated to continue*.' [P6]
Administration	'*I get the impression there was a lot on paper and it was very administratively heavy and it wasn't ideal.*' [P17] '*Digitalisation of the ISF* [Investigator Site File] *is something that we are aiming, since we know that a lot of sites will benefit from it. However, it will be a time intensive process till we achieve this…'* [P21]
**Study team**	
Clinical team	'*I only have a sidenote, that I was strongly supported by excellent research nurses*.' [P5] '*The team was very motivated, it was just and I'm sure there are things that you know if you think about it, you think I would have done that differently. But also I think with the setting in the background and the ways of COVID, it was a time that was very difficult. And even you know, with a very experienced team, maybe it wouldn't work.*' [P7]
Sponsor team	'*I think probably it was very challenging because the sponsor’s team was inexperienced for the kind of study that it was. Personally, I think the study was probably under-resourced, which made it even more challenging for the team*.' [P17] '*It must be incredibly difficult to find someone and to find the perfect person at short notice in the middle of a pandemic.*' [P18] *'I feel that the financial follow up management on the sponsor side was suboptimal and then I think it also made it difficult for us to follow.*' [P17]
External parties	'…*in such studies it is important to ask and know what is each person’s role. A task-responsibility matrix like we have in an interventional study can be handy*.' [P18]
**Decentralised model**	
Recruitment	'*That’s not simple, is it? Because if you have to organise that in a hospital, you have to just reserve a room, take care that the right people are there, in terms of computers, in terms of everything… but here, have to hustle and you have to hit the road…*' [P5] '…*as a GP, being able to perform the inclusions via Teams only and being able to schedule it myself when it was convenient, was a big advantage in terms of time investment.*' [P20] '*In the future, we should try to organise everything more remotely, if of course the legislation can allow it. But I think the example of the United Kingdom, where patients can almost completely sign up for participation online by themselves, is a good example*.' [P12]
**Participants’ views and opinions**	
Trial participation	'*There was a good followup when the nurse said that he should call me the next week. Then he called me the next week so I was never left alone.*' [P15] *'Both the nurse and the doctor were helpful. I did not mind that the doctor was not my family doctor as I felt comfortable. Personally, I do not believe that my own GP would have done anything more.*' [P2] '*After my participation in the trial ended, I went in a blood transfusion centre to donate blood, and there I was informed that I cannot give blood for a year after I participated in the study. Now that was something for maybe was in the information form, but anyway, I was surprised that there was a direct link between my participation in the study and the blood transfusion centres.'* [P12]
Boosting recruitment	'…*advertising more through television might have brought more participants. During the lockdown, people were watching TV more than usually, so that could have potentially brought more patients*.' [P19] '*Having more posters in the test centres would have been informative. I was informed by the nurse at that time, but I do not recall seeing any posters*.' [P2] '*I think that doctors are the best way of spreading the word. There is a well-established bond with the patient, which cannot be compared with any other sort of advertisement.*' [P15]

**Table 4.** Themes and subthemes for domain 'mechanisms of impact'

Themes and subthemes	Illustrative quotations
**Recruitment**	
General practice setting	'…*incidence at practice level was too low to really do the study. I think the set up at practice level and the organisation at the level above was not present to accommodate this study*.' [P4] '*As a participating GP, I was only one day in the week at the practice, and that’s too little to run the research there…*' [P5]
Recruitment process	*'I think a more experienced project manager may have piloted some of the recruitment approaches in a smaller scale and then assessed whether they thought they were going to work or not or how you needed to change them.'* [P18] *'We don't know what difference it would have made, but you could have the automated messages, the text messages you get that you know your test result is positive. If it could have said you know studies are open, go here...'* [P17]
Regional differences	'*Regional differences can be attributed to the fact that Wallonia has a more hospital-based care, and also we, in Flanders, receive quite a considerable research formation during our training as GPs*.' [P8] '*The unregistered product entailed risks that made general practitioners* [in Wallonia] *say that they we don't want to participate in this study because, they are already afraid for our patients. We don't know what the disease is and on top of that we are afraid to prescribe a new drug*.' [P10]
**Research environment**	
Research infrastructure in general practice	'*I think a change in infrastructure is needed. What must be always said here is that with infrastructure we mean infrastructure in terms of staffing… in terms of people, and not in terms of bricks or buildings or whatever or even machines. We need to build infrastructures for GPs to use.'* [P5] *'I'm not sure how many future studies are going to be similar to the DAWN. I think if we're talking about GP studies, I think having more and more GPs who are experienced in research will help.*' [P9] '*Having a CTC* [Clinial Trial Centre] *specialised in general practice*.' [P8]
Government involvement	'*For medication and seroprevalence studies, the involvement of the government in making the trials more known should be established. This can help us have a better recruitment in general practice practices.*' [P2] '*I think it’s a real shame that the therapeutics task force wasn't able to offer more support in communication.*' [P17]

### Pandemic context

Interviewees mentioned the rapidly changing pandemic context as the main challenge. This included uncertainty on the evolution of the pandemic, COVID-19 incidence and severity, and overall risks of a trial for a relatively new illness.

The start of the trial was felt to be too late because vaccinations had already begun. This, in addition to the study halts, gave the impression of being behind the curve. Nonetheless, responders felt that there was still little time for the team to plan and pilot the trial properly.

In addition, responders mentioned that the trial was hampered by the disruption to routine care: clinical services were in constant flux and overstretched. General practice was overwhelmed and to some extent bypassed for the care of COVID-19 patients.

Finally, the public opinion on COVID-19 had changed by the time the trial started. All Belgian residents were invited for vaccination from 28 December 2020, resulting in 30% full vaccination rates (at least two doses) by 20 June 2021.^4^ People were no longer interested in participating in a trial for a disease that they felt would not make them very sick and they were tired of COVID-19 (Table 2).

### Implementation

The design of the trial was felt to be innovative in response to the unique pandemic situation. Decentralised recruitment with the support from study nurses was a strong point, allowing GPs to fit in the study in their busy schedules. On the other hand, the overall setup was still not sufficiently adapted to the general practice context and too much based on hospital trials where clinical staff are linked to a particular site, which is at odds with the decentralised model. Recruitment in general practice was also considered to be more complex than in hospital-based studies.

Training was mostly online because of COVID-19 restrictions, hindering some GPs' ability to take all information on board. The burden of administrative processes and site monitoring was a reason for GPs to stop participation. More procedures could have been done remotely and digitally rather than in person and on paper. The frequent pauses in recruitment demotivated some GPs.

The clinical and sponsor team were too inexperienced according to the responders. The large number of external parties led to role confusion and delays.

Participants felt well supported in the decentralised model. Study nurses especially were considered particularly helpful and trustworthy. The decentralised model could have been advertised more strongly, including national tv advertisements, posters in test centres, or by people’s own GP (Table 3).

### Mechanisms of impact

Recruitment in practices was difficult because of the low incidence per practice. GPs did not have enough time to really engage with the trial. Other recruitment processes were tried but not piloted. GPs also expressed concern on trying an unknown drug in a general practice setting for a new illness.

For future trials, research infrastructure was considered the way forward. Research infrastructure includes experienced research staff in practices who can support clinicians in research. Gaining experience with trials in general practice was also important for future studies to succeed. Finally, responders felt the government and governmental bodies, such as the COVID-19 Task Force, could have done more to support the trial considering they were instrumental in the trial conception and funding (Table 4).

## Discussion

### Summary

The DAWN antivirals trial was set up when there was still an urgent need for effective treatment of COVID-19 patients in general practice. Recruitment was, however, insufficient and the trial was subsequently halted after 13 months. There were 12 months between trial conception and the actual start, largely due to the lengthy pre-submission advice process, delays to trial medication, and difficulties in finding staff. As a result, the trial opened when vaccination campaigns were well advanced, leading to a lower risk of serious illness and a subsequent change in endpoint, but also to decreased interest in a trial for a disease that was no longer considered a major threat.

The pandemic itself had a huge impact on the trial: recruitment settings changed from testing centres to general practices to a decentralised system with remote informed consent; general practice was overwhelmed; study nurses were in short supply and charging very high rates; and the number of potential participants was highly dependent on virus circulation in the population.

The sponsor team was small, inexperienced, recruited specifically for this trial, and experienced a high turnover. This trial required five successive major protocol amendments in 12 months, creating a high workload for the sponsor and clinical teams.

### Strengths and limitations

The DAWN antivirals trial was a randomised placebo-controlled trial with a patient-relevant outcome. The trial was the first randomised controlled trial in Belgium with an unregistered product in general practice. Regulatory authorities were reluctant to grant approval and additional checks were requested to safeguard participants, which had an impact on the team and budget.

The authors developed a decentralised model that allowed participation from people across the country without geographic restrictions. However, such a model was very dependent on awareness of the trial and expensive in terms of study nurse time when home visits are required. Creating such awareness may require national campaigns using multiple public media channels and support from other clinical services such as contact tracing centres.

The authors interviewed 21 stakeholders and trial participants, ensuring a variety of perspectives and experiences to be included in the qualitative study. In the interest of time and budget, the authors were not able to analyse the data with two independent researchers. Another limitation was that no participants from Wallonia could be interviewed because none accepted the invitation. In addition, because of lack of consent, the authors were not able to invite to interview potential participants who had declined to take part in the trial.

### Comparison with existing literature

To date, the largest randomised trial investigating the efficacy of novel therapeutic agents against COVID-19 in the community is the UK-based PANORAMIC trial.^8^ This trial of more than 25 000 participants evaluating molnupiravir was able to build on previous study experience, including the Principle trial,^9^ the ALIC^4^E trial,^10^ and the UK’s strong general practice research infrastructure. Principle was a platform trial evaluating six treatments for COVID-19 in the community: inhaled budesonide, azithromycine, doxycycline, colchicine, favipiravir, and ivermectin in almost 12 000 participants. Pending results for the last two evaluations, only inhaled budesonide has been found to be effective.^9^ Both PANORAMIC and Principle were spearheaded by a large team with vast experience and supported by a large number of other research groups across the country. In addition, they were able to tap into the long-established research infrastructure in the clinical setting to help clinicians with trial procedures. Recruitment was fully remote, with participants conducting all study procedures themselves and data collection either online or via telephone. The trials also used clinical data to contact potential participants proactively.^11^

International collaboration may overcome some of the barriers of general practice trials, by increasing the number of sites and therefore recruitment rates. In general practice, international collaboration has been shown to be effective, for example in the ALIC^4^E trial, which recruited in 15 European countries to evaluate oseltamivir in patients with flu-like illness in pre-COVID times.^12^ However, researchers from across Europe explored collaborations with the PANORAMIC trial, but none were successful in securing funding. The TOGETHER trial recruited COVID-19 outpatients for the evaluation of 10 treatments to date in 22 general practice sites in Brazil and later in South Africa and Pakistan (www.togethertrial.com),^13,14^ supported by research groups from six countries across the world (Brazil, Canada, Pakistan, Rwanda, South Africa, and US). This platform trial has evaluated five treatments so far (hydroxychloroquine, lopinavir/ritonavir, fluvoxamine, ivermectine, and metformin), of which only fluvoxamine was found to be effective.^14^ However, it should be noted that although this trial was designed and conducted by an international group, recruitment was restricted to Brazil in the first instance, suggesting that some countries are better suited than others because of factors such as regulatory processes, incidence of disease, and clinical services.

Regulatory bodies were reluctant to grant approval for a study with an unregistered product in general practice, even when the unregistered product (camostat) had been in use for more than three decades in Asia with an excellent safety profile. This chimes with the experience in the COVERAGE trial, a platform trial conducted in France evaluating telmisartan, inhaled ciclesonide, and interferon ß–1b, where stakeholders felt that regulatory processes were not well adapted to the general practice context.^15^ Trials in general practice could benefit from the creation of a general practice Clinical Trial Centre, where all regulatory processes are controlled and general practice expertise is developed.

### Implications for research

Conducting trials in a general practice context is challenging, especially during a pandemic. Comparing the authors' experience with that of other trials that did manage to recruit to target and report study findings, it appears that large study teams with trial experience, established research infrastructure, and regulatory processes that are adapted to the context are pivotal.

Such a research infrastructure should be tailored to the specific needs of general practice, in that it should be present in the practices and staffed by experienced trial personnel, such as research nurses and dedicated study staff who can dedicate time to research. IT services should allow maximum digitalisation of data processes. Where possible, remote procedures should be adopted to allow participants from other practices to join the study without limitations. Pre-established research infrastructure may already overcome some of those barriers and increase expertise in both sponsor and clinical teams. This will require funders to acknowledge that general practice research comes with its own complexities that should be funded appropriately.

Another shared trait of successful trials during the pandemic is the use of a platform design. Platform trials such as COVERAGE, the TOGETHER trial, and Principle have the advantage that they are able to add new treatments relatively easy and stop treatments that do not work.^16^ Existing platform trials, such as the REMAP-CAP trial^17^ have pre-established structures and procedures in place, which allowed them to quickly enrol COVID-19 patients, even when the platform trial was originally set up for a different target condition (in this case, community-acquired pneumonia). Researchers and funders could draw on the PEARL barriers (political, ethical, administrative, regulatory, and logistical) that were identified by the PREPARE consortium (www.prepare-europe.eu). Solutions to these barriers include rapid access to funding, pre-identification of research questions, the development of generic study methods in master protocols, standardised case definitions, and consent forms in pre-approved protocols, which would expedite the set-up of clinical studies.

The process evaluation of the DAWN antivirals trial has identified strengths, weaknesses, and challenges: the pandemic itself, with an impact on clinical services, staff, IMP delivery, and budget; disease severity changing dramatically over the course of the trial due to widespread vaccination; limitations of conventional recruitment models in general practice; a decentralised recruitment model that allowed all potential participants to self-refer but required awareness among the general public; and a lack of expertise in general practice trials using an unregistered product, both on the sponsor side and at the regulatory bodies. Developing research infrastructure in general practice could overcome many of these hurdles for future studies.

## Data

The dataset relied on in this article is available from the corresponding author on reasonable request.
